# The effect of lifestyle intervention in obesity on the soluble form of activated leukocyte cell adhesion molecule

**DOI:** 10.1186/s12902-016-0138-2

**Published:** 2016-10-13

**Authors:** Alba Sulaj, Johanna Zemva, Ulrike Zech, Annika Woehning, Maik Brune, Gottfried Rudofsky, Peter P. Nawroth, Thomas Fleming, Rüdiger von Bauer

**Affiliations:** 1 Department of Internal Medicine I and Clinical Chemistry, University of Heidelberg, Heidelberg, Germany; 2 German Center for Diabetes Research, Neuherberg, Germany; 3 Institute for Diabetes and Cancer IDC, Helmholtz Center Munich and Joint Heidelberg-IDC Translational, Diabetes Program, University of Heidelberg, Heidelberg, Germany

**Keywords:** Obesity, Lifestyle intervention, Insulin resistance, sALCAM, Cell adhesion molecule

## Abstract

**Background:**

The aim of this study was to investigate the effect of a lifestyle intervention in obesity on the soluble form of the activated leukocyte cell adhesion molecule (sALCAM) and its association with metabolic parameters.

**Methods:**

Twenty-nine obese subjects selected from the OPTIFAST®52 program. This program consisted into 2 crucial phases: an initial 12-week active weight reduction phase, followed by a 40-week weight maintenance phase. At baseline, after 12 weeks and at the end of the program, fasting glucose and insulin, total cholesterol, LDL-C, HDL-C, triglycerides, adiponectin, leptin, high sensitivity CRP, sALCAM, homeostasis model assessment-estimated insulin resistance (HOMA-IR) and leptin-to-adiponectin-ratio were determined. Oral glucose tolerance test (OGTT) was performed when indicated.

**Results:**

At baseline, the serum concentration of sALCAM was increased and correlated positively with HOMA-IR and negatively with age. At the end of the program, sALCAM concentrations decreased significantly. Multivariate analysis showed that sALCAM significantly correlated with age, glucose concentration after 2 h OGTT and the HOMA-IR. A higher decrease of HOMA-IR during the study was observed in subjects with higher concentration of sALCAM at baseline.

**Conclusions:**

sALCAM might be a novel biomarker in obesity that correlates and predicts insulin sensitivity improvement and that can be affected by lifestyle intervention.

## Background

Obesity is considered a major risk factor for cardiovascular diseases, type 2 diabetes, several cancers as well as musculoskeletal disorders [1]. There is agreement within the field that chronic systemic inflammation is associated with insulin resistance in obesity [2]. However a molecular mechanism that explains the causality between the two, particularly within the context of obesity, has yet to be established. In this respect, new biomarkers that can predict inflammation and insulin resistance in obesity need to be further investigated.

Recently, it was demonstrated in a model of delayed type hypersensitivity, that the activated leukocyte cell adhesion molecule (ALCAM/CD166) promotes inflammation by binding the danger-associated molecular pattern (DAMP) molecule, S100B leading to activation of NF-κB [3]. ALCAM showed close structural homology with the receptor for AGEs (RAGE) and it was shown that each receptor could functionally substitute the other in response to S100B [3]. It has previously been shown that an inverse association exists between the soluble form of RAGE (sRAGE) and body mass index (BMI) [4].

Analogous to sRAGE, sALCAM represents the proteolytic cleavage of the ectodomain of the full receptor by “A Disintegrin and Metalloproteinase Domain 17/Tumor Necrosis Factor α Converting Enzyme” (ADAM17/TACE) [5]. The function of sALCAM has been previously studied by means of recombinant isoforms and ALCAM-IgG-Fc chimeras, which were able to inhibit cell-cell adhesion and to promote and/or inhibit cell migration and has been proposed as a biomarker in several cancers [6, 7]. In vitro studies have shown that sALCAM is involved in ALCAM-dependent and –independent immune responses upon inflammatory stimulation and that endothelial expression of ALCAM is induced by hypercholesterolemia [8, 9]. This would suggest sALCAM as a potential biomarker of the innate immune system in inflammation-associated disorders such as hypercholesterolemia, diabetes and obesity.

The aim of this study was to investigate the metabolic role of sALCAM in severely obese patients and whether a 52-week lifestyle intervention program would modulate sALCAM serum concentration.

## Methods

### Study population

This study involved 29 subjects (18 women and 11 men) randomly and retrospectively selected from the OPTIFAST®52 program (franchise holder Nestlé Inc., Switzerland) at the University Hospital of Heidelberg. The OPTIFAST®52 program is a 52-week lifestyle intervention that has been previously described [10]. For the purpose of this study, patient samples and data were studied at three time-points: at the beginning of the program (T0), after 12-week of active weight loss phase through a low-calorie formula-diet (T1) and after 40-week of weight maintenance phase (T2). Subjects had the following comorbidities: hypertension (*N* = 15), insulin resistance (*N* = 25), arthrosis (*N* = 7), substituted hypothyroidism (*N* = 6), hyperlipidemia (*N* = 4), Type 2 Diabetes mellitus (*N* = 3), obstructive sleep apnea syndrome (*N* = 3), non-alcoholic fatty liver disease (*N* = 3) and anemia, chronic obstructive pulmonary disease, hyperthyroidism, polycystic ovary syndrome (each *N* = 1). With respect to medication, 18 subjects were under antihypertensive medication, whereas ten subjects took oral antidiabetic drugs, three were under insulin treatment and four were taking statins. Written informed consent was given by participants for their clinical records and blood samples to be used in this study. This full study and the use of archival material from patients were specifically approved from the “Ethics Committee” of the Medical Faculty of the University of Heidelberg in compliance with national guidelines and the declaration of Helsinki. Full clinical characteristics of the subjects at baseline are given in Table 1.

**Table 1:** Clinical characteristics of patients at baseline

Characteristics	Value (*n* = 29)
Age (years)	42.3 ± 2.2
Gender (female/male)	18/11
BMI (kg/m^2^)	46.4 ± 1.7
History of:	
Hypertension (yes, n [%])	15 [50]
Insulin resistance (yes, n [%])	25 [83]
Diabetes Mellitus Type 2 (yes, n [%])	3 [10]
Hypercholesterolaemia (yes, n [%])	4 [13]
Allergies (yes, n [%])	6 [20]
Arthrosis (yes, n [%])	7 [23]
Hypothyroidism (yes, n [%])	6 [20]
Nonalcoholic Fatty Liver Disease (yes, n [%])	3 [10]
Obstructive Sleep Apnea (yes, n [%])	3 [10]
Medication:	
Antihypertensive (yes, n [%])	18 [60]
Oral hypoglycemic (yes, n [%])	10 [33]
Insulin (yes, n [%])	3 [10]
Statins (yes, n [%])	4 [13]

Data represent mean ± SE

### Anthropometrical and laboratory measurements

Height, weight and blood pressure were measured regularly using standard method. Body mass index (BMI) was consistently calculated by the formula: BMI = Weight (kg)/Height (m)^2^. Blood samples were collected at described time-points (T0, T1 and T2) and immediately analyzed. Serum samples were stored at −80 °C for further analysis. Fasting glucose and insulin, total cholesterol (T-Cholesterol), low density lipoprotein (LDL), high density lipoprotein (HDL), triglycerides, adiponectin, leptin and high sensitivity CRP (hsCRP) levels were measured in the central laboratory of the University Hospital of Heidelberg. The oral glucose tolerance test (OGTT) was performed at the Endocrinology Ambulance of the University Hospital of Heidelberg and glucose level after 2 h was measured at the central laboratory. Homeostasis model assessment-estimated insulin resistance (HOMA-IR) was calculated by the formula: HOMA-IR = fasting Glucose [in mg/dl]*fasting Insulin [in mU/l]/405. Leptin-to-Adiponectin Ratio (LAR) was calculated by the formula: LAR = Leptin/Adiponectin. The serum concentration of sALCAM was determined by enzyme linked immuno-sorbent assay (ELISA) (DY656, *R&D Systems, USA*) according to the manufacturer’s protocol with a sample dilution of 1:800 in triplicate.

### Statistical analysis

SPSS version 20.0 (*IBM SPSS, Inc. Armonk, NY*) and GraphPad Prism version 5.00 for Windows (*GraphPad Software, San Diego California USA*) were used to analyze the collected data. Where indicated, values are given as mean and SE. Due to small sample size, normal or other exact distribution could not be specified, therefore non-parametric statistical tests were applied. Univariate Spearman’s rank correlation coefficient (R) was used for correlation analysis at baseline. Friedman test was used for overall comparison between measurements and where significant Wilcoxon signed-rank test was applied for comparing single point measurements. Repeated measurements of identified parameters in different subjects were analyzed as panel data. Therefore a multivariate linear regression model for panel data was created by the stepwise inclusion of all explanatory parameters of sALCAM level, considering also joint effects of parameters. Results were expressed as standardized correlation coefficient (*β*) under a 95 % confidence interval. The likelihood-ratio test on the significance of the model was calculated. Collinearity analysis was also performed. Mann-Whitney test was applied to compare sALCAM mean level at baseline among the quartiles of changes in HOMA-IR (ΔHOMA-IR). Values of *P* < 0.05 were considered statistically significant.

## Results

A total of 29 subjects (18 female and 11 male) were part of this study (Table 1). At baseline, subjects were severely obese with a mean BMI of 46.4 ± 1.7 kg/m^2^. Half of the subjects suffered from hypertension with a MAP of 109 ± 2.6 mmHg and were treated accordingly with lowering blood pressure medication. With respect to glucose metabolism, 3 of the subjects had diabetes mellitus type 2. The remaining 48 % had impaired fasting glucose and 12 % had impaired glucose tolerance according American Diabetes Association (ADA) criteria [11]. 83 % of patients had insulin resistance, according to the HOMA-IR with a cut-off value of 2.3 [12]. Adiponectin levels were decreased, whereas leptin and LAR were increased (4.8 ± 0.4 μg/ml, 54.1 ± 5.9 μg/l and 12.7 ± 1.4 respectively), which are considered predictors of insulin sensitivity [13, 14]. hsCRP levels at baseline were increased as a sign of increased non-specific inflammation (Table 2).

**Table 2:** Anthropometric and metabolic characteristics of patients during the study

Parameter	T0	T1	T2	Friedman Test	P(T0-T1)	P(T1-T2)	P(T0-T2)
Age (years)	42.3 ± 2.2						
Gender (female/male)	18/11						
BMI (kg/m^2^)	46.4 ± 1.7	38.3 ± 1.4	36.3 ± 1.4	***	***	*	***
MAP (mmHg)	109.0 ± 2.6	93.4 ± 2.3	103.5 ± 2.4	***	***	*	n.s.
Fasting glucose (mg/dl)	110.2 ± 7.0	90.7 ± 2.1	89.7 ± 2.2	***	***	n.s.	***
2 h-Glucose OGTT (mg/dl)	117.9 ± 6.6	117.5 ± 4.7	93.2 ± 3.6	***	n.s.	***	***
HOMA-IR	7.8 ± 1.2	3.3 ± 0.3	3.8 ± 0.5	***	***	n.s.	**
T-cholesterol (mg/dl)	192.2 ± 7.6	157.4 ± 5.5	181.0 ± 6.8	***	***	***	n.s.
LDL (mg/dl)	110.3 ± 6.4	98.3 ± 4.5	105.8 ± 5.3	*	**	n.s.	n.s.
HDL (mg/dl)	53.4 ± 4.4	38.9 ± 1.9	53.8 ± 2.6	***	***	***	n.s.
Triglycerides (mg/dl)	149.5 ± 22.9	100.8 ± 5.2	123.9 ± 30.9	**	***	n.s.	**
Adiponectin (μg/ml)	4.8 ± 0.4	6.7 ± 0.5	7.9 ± 0.7	***	***	*	***
Leptin (μg/l)	54.1 ± 5.9	20.5 ± 2.7	32.9 ± 5.8	***	***	**	***
LAR	12.7 ± 1.4	3.5 ± 0.5	5.7 ± 1.1	***	***	*	***
hsCRP (mg/l)	10.1 ± 2.1	6.1 ± 1.1	3.4 ± 0.8	***	*	**	***

Friedman test was used for overall comparison and where significant Wilcoxon signed test was used for comparison between single point measurements. Data represent Mean ± SE. *P < 0.05, **P < 0.01, ***P < 0.001, n.s. = non-significant

All the subjects tolerated the lifestyle intervention program well. At the end of the study, whilst decreased significantly by 22 %, the mean BMI was 36.3 kg/m^2^, which would still be classified as Obesity class II, according to WHO criteria. However the metabolic parameters had improved significantly after 52 weeks (Table 2). Glucose metabolism and insulin sensitivity were improved and the dose of medication taken was reduced in 73 % of the subjects treated with oral antidiabetic drugs. MAP decreased significantly after 12 weeks and at the end of the study the antihypertensive medication was reduced in 78 % of the subjects treated. Half of the subjects treated with lipid lowering agents had also reduced their medication. The respective medications were reduced starting from week 12. These results are consistent with previous studies on similar lifestyle intervention programs [15].

**Fig. 1:**
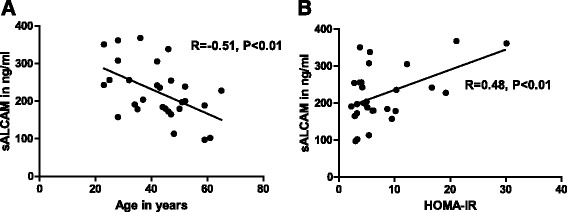
Correlation of serum concentration of sALCAM with Age and HOMA-IR at baseline. Serum concentration of sALCAM correlated negatively with age (**a**) and positively with HOMA-IR at baseline (**b**). Univariate Spearman’s rank correlation coefficient (R) was used for correlation analysis. Values of *P* < 0.05 were considered statistically significant

**Fig. 2:**
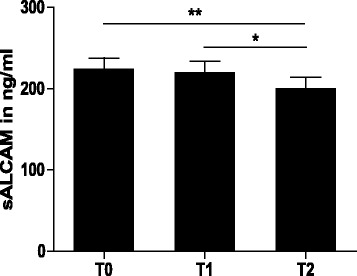
Serum concentration of sALCAM during the study. sALCAM serum concentration decreased significantly at the end of the program. Friedman test was used for overall comparison between measurements and where significant Wilcoxon signed-rank test was applied for comparing single point measurements. *P < 0.05, **P < 0.01

**Fig. 3:**
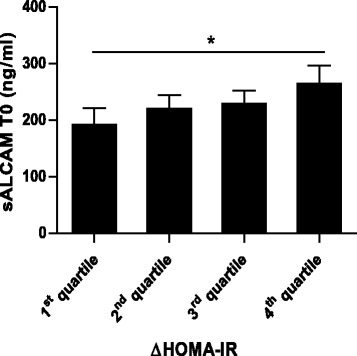
sALCAM serum concentration at baseline and decrease of HOMA-IR (ΔHOMA-IR). Subjects who showed a higher decrease of HOMA-IR had higher serum concentration of sALCAM at baseline. Data are shown as quartiles of the reduction of HOMA-IR (ΔHOMA-IR) and mean levels of sALCAM at baseline with error bars showing SE. Wilcoxon signed-rank test was applied for comparing single point measurements. *P < 0.05

sALCAM serum concentration at baseline was increased when compared to serum concentration of sALCAM in healthy subjects, reported in previous studies [5, 6]. sALCAM levels at baseline correlated negatively with the age of the subjects (*R* = −0.51. *P* < 0.01) (Fig. 1a) and positively with the HOMA-IR (*R* = 0.48, *P* < 0.01) (Fig. 1b) whereas no correlation between sALCAM and BMI was observed (*data not shown*). At the end of the program, the concentration of sALCAM had decreased significantly by 11 % as compared to baseline (224.0 ± 13.7 ng/ml vs. 199.5 ± 14.0 ng/ml) (Fig. 2). Multivariate analysis for panel data showed that during the study serum concentrations of sALCAM significantly correlated with age (β = −3.44, *P* = 0.01), glucose concentration after 2 h OGTT (2 h-OGTT, β = 0.37, *P* = 0.008) and the HOMA-IR (β = 1.52, *P* = 0.04) (Table 3). However, sALCAM levels did not correlate significantly with BMI, LAR and hsCRP and all joint effects of the parameters were found to be non-significant. The likelihood-ratio test of the model was significant (LR = 590, df = 15). Collinearity analysis showed no significant multicollinearity (VIF < 3). Furthermore, baseline levels of sALCAM were compared among subjects with differences in the HOMA-IR at the end of the study. For this purpose subjects were divided according to the quartiles of the reduction of HOMA-IR between baseline and end of the program (ΔHOMA-IR). Baseline levels of sALCAM were found significantly higher in subjects with reduction of HOMA-IR belonging to the fourth quartile when compared to those belonging to the first quartile (191.6 ± 30.1 ng/ml vs. 254.1 ± 29.1 ng/ml, *P* < 0.05) (Fig. 3). This would suggest sALCAM level at baseline as a biomarker for predicting the metabolic effect of lifestyle intervention programs on improving insulin sensitivity. When age in between the two groups was compared it was found that the subjects with the higher HOMA-IR improvement and higher sALCAM levels at baseline were younger, as it is excepted since sALCAM level correlated negatively with age (37 ± 2,3 years vs. 50 ± 3 years, *P* = 0,005).

**Table 3:** Multivariate linear regression analysis for sALCAM as dependent variable through the study

Independent variable	sALCAM	
	*β* Coefficient (95 % CI)	*P* value
Age	**−3.44 (−5.95:−0.92)**	**0.01**
BMI	−0.53 (−2.46:1.40)	0.58
2 h- OGTT	**0.37 (0.11:0.64)**	**0.008**
HOMA-IR	**1.52 (0.01:3.02)**	**0.04**
LAR	0.47 (−0.89:1.83)	0.48
hsCRP	0.35 (−0.96:1.67)	0.58

Multivariate linear regression model for panel data was created by the stepwise inclusion of explanatory parameters of sALCAM level, considering also joint effects of parameters. Results were expressed as standardized correlation coefficient (*β*) under a 95 % confidence interval. Values of *P* < 0.05 were considered statistically significant and are presented in boldface

## Discussion

In this study, the effect of weight reduction in severely obese patients on serum sALCAM, a soluble isoform of a recently described PRR, was investigated. It was shown that sALCAM was increased in severely obese patients and decreased significantly during a lifestyle intervention program. The levels of sALCAM measured in this study were higher when compared to levels of sALCAM in healthy subjects measured in previous studies [5–7]. sALCAM represents the proteolytic cleavage of the full receptor and has been proposed as a biomarker for different cancers [5–7]. In the context of obesity there is lack of studies on the potential role of sALCAM. In previous work by this group it was found that serum sALCAM is increased in subjects with metabolic disorders, such as type 2 diabetes and familiar hypercholesterolemia (*data in review*). Increased shedding of the receptor could indicate a protective mechanism where sALCAM acts as a decoy receptor by inhibiting ligand binding to the transmembrane glycoprotein ALCAM and therefore preventing its activation. Similiar function was observed in previous work in our group regarding scavenging of RAGE ligands by sRAGE which led to reduced delayed-type hypersensitivity even in mice lacking RAGE. In this way we were able to identify ALCAM as a close functional and structural homologue of RAGE [3]. Further studies are required to determine whether the scavenging of ALCAM or RAGE ligands by sALCAM is functionally related to the inhibition of detrimental ALCAM signalling in obesity. Due to the relative low affinity of the ligands to their respective receptors however, it seems unlikely since in vivo levels of the ligands are significantly lower than the amount required in vitro for their physiological function [3]. This could suggest that sALCAM functional role might be primarily in local tissue rather than in serum and future studies are needed to investigate tissue expression of sALCAM and sALCAM-ALCAM interaction in obesity.

No correlation was observed between sALCAM and BMI during the course of study. However previous studies have shown that BMI is not a sensitive marker for obesity-related risk of metabolic disorders [16, 17]. Non-obese subjects with metabolic disorders and obese subject with no obesity-associated metabolic complications have already been published [18]. Instead of BMI other markers, such as those of obesity-induced insulin resistance have been investigated extensively [19, 14].

In this study it was shown that sALCAM serum concentration correlated significantly with the HOMA-IR as a well-established marker of insulin resistance but no correlation was observed with the levels of hsCRP, as a nonspecific systemic marker of inflammation [19, 13, 20]. It has previously been reported in primates that endothelial expression of cell adhesion molecules, such as VCAM-1 and P-Selectin, is an early event in diet-induced obesity that occurs simultaneously with the development of insulin resistance but does not correlate with systemic markers of inflammation, such as CRP [21]. Whether the expression of the full receptor ALCAM in endothelial cells or circulating cells such as macrophages is involved in the molecular mechanism of obesity-induced insulin resistance requires further investigation. However it was shown in this study that shedding of ALCAM into the serum of obese patients predicts the metabolic success of weight reduction with respect to insulin sensitivity. In this regard pure chronological age effect in the improvement of insulin sensitivity is excluded, since previous study has showed that it is increased adiposity related to aging that explains the reduction in insulin sensitivity rather than chronological age itself [22].

A limitation of this study is the small sample of subjects recruited. We acknowledge that the model we propose explains only the variables that were considered and there might be other factors influencing the levels of sALCAM. However the considered variables explain significantly part of the changes in sALCAM level. Unexplained changes due to non-included factors need to be further investigated.

Future experimental analysis should provide new insights on the functional role of sALCAM in particularly its involvement in ALCAM-dependent and –independent immune responses in low-grade inflammatory state and whether the scavenging of ALCAM ligands by sALCAM is functionally related to the inhibition of ALCAM signalling.

## Conclusions

This study identifies for the first time sALCAM as a novel biomarker in severe obesity. Serum concentration of sALCAM was not only increased in severely obese subjects but also decreased significantly through a lifestyle intervention program. Moreover, serum sALCAM levels correlated positively with levels of HOMA-IR and predicted the success of the program in improving insulin sensitivity. sALCAM might be a novel biomarker of immune responses in obesity which can be significantly affected by lifestyle intervention programs.

## Abbreviations

ADAM17/TACE A: Disintegrin and Metalloproteinase Domain 17/Tumor Necrosis Factor α Converting Enzyme
ALCAM: Activated leukocyte cell adhesion molecule
BMI: Body-mass index
ELISA: Enzyme linked immuno-sorbent assay
HDL: High density lipoprotein
HOMA-IR: Homeostasis model assessment-estimated insulin resistance
hsCRP: High sensitivity CRP
LAR: Leptin-to-Adiponectin Ratio
LDL: Low density lipoprotein
MAP: Mean arterial pressure
OGTT: Oral glucose tolerance test
RAGE: Receptor for advanced glycation end products
sALCAM: soluble form of ALCAM
sRAGE: soluble form of RAGE
T-Cholesterol: total cholesterol

## References

[1] WHO (2000). Obesity: preventing and managing the global epidemic. Report of a WHO consultation. World Health Organ Tech Rep Ser.

[2] Khodabandeloo H., Gorgani-Firuzjaee S., Panahi S. and Meshkani R. Molecular and cellular mechanisms linking inflammation to insulin resistance and β-cell dysfunction. Transl Res. 2015. doi:10.1016/j.trsl.2015.08.011. 10.1016/j.trsl.2015.08.011 26408801

[3] von Bauer R, Oikonomou D, Sulaj A, Mohammed S, Hotz-Wagenblatt A, Gröne HJ, Arnold B, Falk C, Luethje D, Erhardt A, Stern DM, Bierhaus A, Nawroth PP (2013). CD166/ALCAM mediates proinflammatory effects of S100B in delayed type hypersensitivity. J Immunol.

[4] Hagen I, Schulte DM, Müller N, Martinsen J, Türk K, Hedderich J, Schreiber S, Laudes M (2015). Soluble receptor for advanced glycation end products as a potential biomarker to predict weight loss and improvement of insulin sensitivity by a very low calorie diet of obese human subjects. Cytokine.

[5] Carbotti G, Orengo AM, Mezzanzanica D, Bagnoli M, Brizzolara A, Emionite L, Puppo A, Centurioni MG, Bruzzone M, Marroni P, Rossello A, Canevari S, Ferrini S, Fabbi M (2013). Activated leukocyte cell adhesion molecule soluble form: a potential biomarker of epithelial ovarian cancer is increased in type II tumors. Int J Cancer.

[6] Witzel I, Schröder C, Müller V, Zander H, Tachezy M, Ihnen M, Jänicke F, Milde-Langosch K (2012). Detection of activated leukocyte cell adhesion molecule in the serum of breast cancer patients and implications for prognosis. Oncology.

[7] Hansen AG, Arnold SA, Jiang M, Palmer TD, Ketova T, Merkel A, Pickup M, Samaras S, Shyr Y, Moses HL, Hayward SW, Sterling JA, Zijlstra A (2014). ALCAM/CD166 is a TGF-β-responsive marker and functional regulator of prostate cancer metastasis to bone. Cancer Res.

[8] Guerraty MA, Grant GR, Karanian JW, Chiesa OA, Pritchard WF, Davies PF (2011). Side-specific expression of activated leukocyte adhesion molecule (ALCAM; CD166) in pathosusceptible regions of swine aortic valve endothelium. J Heart Valve Dis.

[9] Ikeda K, Quertermous T (2004). Molecular isolation and characterization of a soluble isoform of activated leukocyte cell adhesion molecule that modulates endothelial cell function. J Biol Chem.

[10] Wallmeier D, Winkler JK, Fleming T, Woehning A, Huennemeyer K, Roeder E, Nawroth PP, Friederich HC, Wolfrum C, Schultz JH, Rudofsky G (2013). Genetic modulation of the serotonergic pathway: influence on weight reduction and weight maintenance. Genes Nutr.

[11] American Diabetes Association (2014). Standards of medical care in diabetes--2014. Diabetes Care.

[12] Esteghamati A, Ashraf H, Esteghamati AR, Meysamie A, Khalilzadeh O, Nakhjavani M, Abbasi M (2009). Optimal threshold of homeostasis model assessment for insulin resistance in an Iranian population: the implication of metabolic syndrome to detect insulin resistance. Diabetes Res Clin Pract.

[13] Tschritter O, Fritsche A, Thamer C, Haap M, Shirkavand F, Rahe S, Staiger H, Maerker E, Häring H, Stumvoll M (2003). Plasma adiponectin concentrations predict insulin sensitivity of both glucose and lipid metabolism. Diabetes.

[14] Finucane FM, Luan J, Wareham NJ, Sharp SJ, O'Rahilly S, Balkau B, Flyvbjerg A, Walker M, Højlund K, Nolan JJ, Savage DB (2009). European Group for the Study of Insulin Resistance: Relationship between Insulin Sensitivity and Cardiovascular Disease Risk Study Group) Correlation of the leptin:adiponectin ratio with measures of insulin resistance in non-diabetic individuals. Diabetologia.

[15] Winkler JK, Schultz JH, Woehning A, Piel D, Gartner L, Hildebrand M, Roeder E, Nawroth PP, Wolfrum C, Rudofsky G (2013). Effectiveness of a low-calorie weight loss program in moderately and severely obese patients. Obes Facts.

[16] Klöting N, Fasshauer M, Dietrich A, Kovacs P, Schön MR, Kern M, Stumvoll M, Blüher M (2010). Insulin-sensitive obesity. Am J Physiol Endocrinol Metab.

[17] Twig G, Afek A, Derazne E, Tzur D, Cukierman-Yaffe T, Gerstein HC, Tirosh A (2014). Diabetes risk among overweight and obese metabolically healthy young adults. Diabetes Care.

[18] Scott RA, Fall T, Pasko D, Barker A, Sharp SJ, Arriola L, Balkau B, Barricarte A, Barroso I, Boeing H, Clavel-Chapelon F, Crowe FL, Dekker JM, Fagherazzi G, Ferrannini E, Forouhi NG, Franks PW, Gavrila D, Giedraitis V, Grioni S, Groop LC, Kaaks R, Key TJ, Kühn T, Lotta LA, Nilsson PM, Overvad K, Palli D, Panico S, Quirós JR, Rolandsson O, Roswall N, Sacerdote C, Sala N, Sánchez MJ, Schulze M, Siddiq A, Slimani N, Sluijs I, Spijkerman AM, Tjonneland A, Tumino R, van Der ADL, Yaghootkar H, McCarthy MI, Semple RK, Riboli E, Walker M, Ingelsson E, Frayling TM, Savage DB, Langenberg C, Wareham NJ, RISC Study Group; EPIC-InterAct Consortium (2014). Common genetic variants highlight the role of insulin resistance and body fat distribution in type 2 diabetes, independent of obesity. Diabetes.

[19] Vogeser M, König D, Frey I, Predel HG, Parhofer KG, Berg A (2007). Fasting serum insulin and the homeostasis model of insulin resistance (HOMA-IR) in the monitoring of lifestyle interventions in obese persons. Clin Biochem.

[20] Preciado-Puga MC, Malacara JM, Fajardo-Araujo ME, Wröbel K, Wröbel K, Kornhauser-Araujo C, Garay-Sevilla ME (2014). Markers of the progression of complications in patients with type 2 diabetes: a one-year longitudinal study. Exp Clin Endocrinol Diabetes.

[21] Chadderdon SM, Belcik JT, Bader L, Kirigiti MA, Peters DM, Kievit P, Grove KL, Lindner JR (2014). Proinflammatory endothelial activation detected by molecular imaging in obese nonhuman primates coincides with onset of insulin resistance and progressively increases with duration of insulin resistance. Circulation.

[22] Karakelides H, Irving BA, Short KR, O'Brien P, Nair KS (2010). Age, obesity, and sex effects on insulin sensitivity and skeletal muscle mitochondrial function. Diabetes.

